# On Professor Nussbaum’s Operation of Nerve-stretching

**Published:** 1878-03

**Authors:** Samuel F. Farrar


Chicago Medical Journal

AND

EXAMINER.

Vol. XXXVI.—MARCH, 1878.—No. 3.

### Original Communications.

### ON PROFESSOR NUSSBAUM'S OPERATION OF NERVE-STRETCHING.

By Samuel F. Farrar, M. D.

On the 23d of June, 1872, Prof. v. Nussbaum performed for the first time in the annals of surgery, the operation of nerve-stretching. The patient, a soldier, had been wounded in the elbow and neck by a rifle ball, and upon the neck an abcess formed, which healed in about fourteen days, but in consequence of these injuries such a violent contraction of the left pectoralis major and minor muscles and all the flexors of the left upper-arm, forearm and hand arose, that not even by main force could the fingers and elbow be bent in a straight line. The sensibility in these parts was very much lowered, but not entirely lost, for though pricking of a pin made no impression, yet deep incisions were felt in a very slight degree. When the patient was anæsthetized, as was often tried, then the arm and hand could be drawn out and kept straight, by being bound to a splint, just as long as the patient was without feeling or consciousness, but in a very few minutes after waking the cramps would return with such violence that had not the bindings around the arm and splint been immediately removed, deep wounds in the skin would have resulted from the mere force of the contraction of the muscles. Then in about two hours after the anæsthetic had been given, while the patient was slightly conscious, powerful muscular contractions took place over the whole body, which lasted, however, but a short time and then passed away. Supported by the investigations of Prof. Voigt, he thought these disturbances depended upon an irritation of the four lower cervical nerves in their motor branches, together with lighter disturbances of the sensory roots at their origin in the cord. And as the medicines which act upon the cord appeared to do more harm than good, he determined to try the experiment of stretching the nerve, in the hope of relieving the agony that the patient suffered. He conceived the possibility of the result from observations made after resection of the elbow-joint, in cases where the ulnar nerve had become stretched, which proved to him that the cramps which took place before the operation, were relieved by the drawing out of the nerve.

The operation was done in this manner: first the patient was anæsthestized to the fullest extent, then a long incision was made directly over the ulnar nerve, then the nerve was carefully dissected out, stretched and put back in its place, the wound carefully washed and sewed up. A second incision was made, just over the axillary artery, and all the thick nerve fibres around the artery were removed from their adhesions to the surrounding tissue, the cutaneous, as well as the muscular, and the median, radial and ulnar distinguished by this, that upon their being stretched, the muscles to which they were distributed contracted violently. This wound was treated in the same way as the other. Finally a third incision was made over the greater curve of the left clavicle, cutting through the platysma myoides, and exposing the inferior cervical nerves which were taken up on the finger and stretched, then each one was followed up by the end of the finger to its foramen and pushed from its egress in all directions; finally a pull was made, as if to separate it from the cord. During these manipulations there were violent contractions of the left arm. Then the lower nerve fibres which in no way were abnormal in appearance were put back as far as possible in their original places, and the wound washed and sewed up.

The result was satisfactory, according to the article in Schmidt's Jahrbücher, which was an extract from a German journal to which I had not access, which contains Prof. Von Nussbaum's own account, written while the patient was still under treatment, though then very much improved. I also find in one of the English journals a short notice of this operation, announcing that it was successful. So the patient may have recovered. The after treatment was nourishment, baths, electricity and exercise. In this case the lesion was due to peripheral irritation, but in another, in which he operated in 1876, it was of centric origin. The patient was a Polish gentleman, who had suffered from paraplegia for eleven years in consequence of a fall, in which severe injuries were received in the sacral region. During this period there had been partial loss of sensation and total loss of voluntary movement. The patient through the whole sickness had been very much tormented by constant tonic spasms, which were at times so severe that the knees were dragged up in front of the chest. The patient was anæsthetized and an incision made along the direction of Poupart's ligament; the anterior crural nerve was taken up and separated from the vein and artery, then the finger passed under and the nerve stretched, when it was returned to its place and the wound closed. The sciatic nerve was treated in the same way, the incision being made between the greater trochanter and the tuberosity of the ischium. At the end of a fortnight the same operations were performed on the other side, and the patient, though not cured, was very much improved, and was able to walk about with crutches, a thing that for years he had not been able to do.

The *Lancet* of June 26th, 1875, gives a case of nerve stretching for neuralgia, which was done by Dr. Callender, at St. Bartholomew's Hospital. The patient was a carpenter, and a little over a year before the operation was done, had had his hand sawed off by a circular saw. At the time that he came to the hospital, he suffered from a painful and unnourished condition of the stump. He had undergone two amputations, before he came, because after the first, the wound did not heal and there was a very painful condition of the forearm, hence the second, a year afterwards, was deemed advisable. This was followed by successful healing, yet the pain still remained, and a few weeks before he came to St. Bartholomew's, he struck the end of the stump, and from that time the pain had greatly increased, and the arm and forearm become cold, the skin glazed and of a dusky color, from want of proper nourishment in the arm. A little while after this, the pain became constant and was always described as being over the region of the median nerve. Every kind of medical treatment suitable for such cases, as morphine, belladonna and anodyne liniments, was tried, but only with a very temporary effect, and the pain meanwhile kept steadily increasing: finally it became so intense, that the patient was unable to sleep and at times became sick and vomited. On the 27th of March, Dr. Callender cut down on the median nerve, which seemed to be thickened in itself, and in its surroundings. After freeing about an inch of the nerve from its adjacent structure, he forcibly stretched it for about three quarters of an inch. Then the wound was closed, and dressed with salicylic acid dressings. On the 25th of April, he was well in every respect, the pain was gone and the natural heat and color of the parts returned.

In this case the irritation may have come from the nerves having been strained in extending the forearm, as, after amputation of this kind, the arm while healing is usually carried flexed, and later when the arm is used and extended, the nerves which have their cut ends attached to the stump or rather the scarred tissues of the stump, resent the strain. There are other ways in which irritations may arise: 1st. If the tissues about a nerve become thickened, and in this way the nerves become bound down to the adjacent structures and so lose their freedom of action, and the power of gliding which belongs to nerves about joints. This is sometimes the case in a fracture of the lower end of the humerus when the recovery takes place with much thickening of the tissues about the internal condyle. Here when the arm comes to be used, it will be found that there is pain in the course of the ulnar nerve, and also numbness in the little and ring fingers whenever the arm is forcibly extended. 2d. The nerves in stumps may become irritated by their having contracted adhesions to the muscles, thus being liable to be pulled or twitched in the movement of these muscles. 3rd. Bands of fibrous adhesions may form across nerves and in contraction of these, pressure on the nerve will arise, and thus cause irritation, or instead of a mere band, dense contractile tissue may surround the nerve for some distance, and so cause irritation of its fibres.

Prof. Vogt reported the following case in the *Centralblatt für Chirurgie* of 1876, and an abstract of this is given in the *British Medical Journal* for 1877. The patient a laborer, aged 63, received an injury of the right hand from a falling stone. At the end of two weeks the palmar wound was entirely healed, and on the dorsal, opposite the lower end of the third metacarpal bone, a healthy granular surface existed ; when trismus set in, severe opisthotonos and clonic convulsions followed, in spite of the free use of opium. There was, however, no tenderness either in the wound or over the course of the nerve in the arm or forearm, but the brachial plexus in the neck was very sensitive, and pressure gave rise to spasm of the muscles. The operation was made by exposing the brachial plexus on the right side of the neck, in the triangle enclosed by the trapezius, omo-hyoid and scaleni muscles, opening its sheath, drawing out and stretching the separate trunks. The sheath appearing strongly injected was loosened from the surrounding tissues, as far as the spinal canal. In the hand, the palmar cicatrix was separated from the sheath of the flexor tendons, by a crucial incision and subsequent dissection, and the cicatrising edge of the dorsal wound excised. Immediately on waking, the patient could open his mouth, and run out his tongue, and all symptoms disappeared except some slight spasms of the muscles of the neck, which followed vomiting on the second day. On the tenth day after the operation, the wounds were nearly healed. The patient received no other medicine than opium for restlessness at night and felt no morbid sensations beyond an occasional pricking in the fingers.

In the *Philadelphia Medical Times* for 1877, the following case of Dr. Ferdimand Petersen is given: The patient was a blacksmith, and while working was struck on the right leg by a piece of steel, the fragment burying itself in the tissues. Persistent pain troubled the patient to such a degree, that he insisted upon having an operation for the removal of the foreign body. Dr. Petersen found the painful spot to be situated in about the middle of the leg on the inside, somewhat back of the tibia. He cut down upon it and explored in various directions, but did not succeed in finding the steel. The nerve which had been laid bare in the operation seemed very sensitive, so Dr. Petersen took it up and stretched it. The wound healed quickly leaving the patient without pain, and able to walk about with comfort, though the fragment of steel remained in the tissue most probably encapsuled.

In one of the last numbers of this same journal, is an account of the stretching of the post-tibial and the popliteal nerves for tetanus caused by the impaction of a splinter beneath the toe, and consequent exposure to cold. Here only a temporary benefit was gained, for though a diminution of the symptoms followed for a short time, the patient finally died. In this case the posterior tibial was injected and thickened; the popliteal normal, and the sciatic nerve exhibited several points of injection. The same article gives another case of nerve stretching by Prof. Vogt for neuralgia, from a wound in the inner part of the right forearm, which, after healing, left impaired movement in the little and ring fingers, attributed to involvement of the flexor tendons in the cicatrix, at the same time the seat of the scar was very painful, especially at one point, which, on being touched, gave rise to acute pain radiating into the fingers. This condition continued for a year, when Vogt dissected out the ulnar nerve from the cicatricial tissue and stretched it in both directions, after which there was disappearance of the neuralgia and complete use of the fingers.

In the *Revue des Sc. Méd.*, *Vol. I*, 1873, is an account of the stretching of the spinal nerves, by Dr. Gartner of Stuttgart, but unfortunately neither the names nor number of the nerves are stated. The patient, a woman aged 38 years, had suffered from right hemiplegia since she was four years old, when suddenly a very acute pain came in the paralyzed arm, accompanied by a sensation of retraction. The nerves were laid bare and stretched by the aid of blunt hooks, the pain disappeared and did not return until death, which followed fifteen days later.

Dr. Drake published in the *Canada Medical and Surgical Journal* for October, 1876, a very long and full case of traumatic tetanus treated in this way.

The patient, a well-nourished, powerful Swede, aged 28, was admitted to the Montreal General Hospital, complaining of a soreness about the throat, difficulty of swallowing, and a great feeling of stiffness. This was on August 25th, and on the 12th, thirteen days previous, he had stepped on a rusty nail, running it through the outer margin of the left foot, an inch above the metatarso-phalangeal articulation of the little toe. He extracted the nail at once, and applied turpentine to the wound. It felt sore for a few days, but as it did not prevent him from working, he did not pay any attention to it; but on the 20th, he noticed that he could not open his mouth as well as usual, when he was taking his food, and that he experienced some difficulty in swallowing, not pain, but a sensation as if the food slipped down too quickly, and with a jerk. Mastication soon became so difficult, that he was obliged to take nothing but liquid food. Also at times, profuse perspiration occurred. These symptoms became daily more marked till the 23d, when he began to feel pain in the back and shoulders, or rather a sense of stiffness, which made it difficult for him to bend, and slight chills came on at night. On the 24th he left work, and although he felt himself growing stiffer and more chilly, he still kept up. On the 25th he was admitted to the hospital, complaining of a soreness in the throat, difficulty of swallowing, and general stiffness, but did not mention the accident he had received, nor, being a foreigner, was he able to give a clear account of himself. In the evening he complained of pain in the chest, back, wrist and ankles, and spent a very restless night, complaining very loudly of wandering pain all over the body. On the 26th, at the morning visit, trismus and opisthotonos were well marked, he was covered with profuse perspiration, complained of intense pain in the back and neck, and had a desire to defecate, but could not. The symptoms rapidly grew worse during the forenoon, when regular tetanic spasms set in, the whole body being convulsed every few minutes. During the spasms the teeth were tightly clenched, the arms and legs drawn up and rigid, and the back arched in permanent spasm, so that he lay with only the back of his head and heels touching the bed. He had great thirst, but could not drink a drop, and the pupils were widely dilated. At 4.30 p. m., the patient was anæsthetized, the sciatic seized with forceps and pulled. The immediate effect was remarkable. Before, and at the time of the operation, the arms and legs were firmly fixed and rigid, the back arched, the head thrown back, and the teeth clenched, although the patient was under chloroform; after the traction was made the limbs became suddenly flacid, the opisthotonos relaxed, the jaws could be opened, and the patient lay quietly on the bed, and without spasm. On coming out of the choloroform, he felt very comfortable, and drank a quantity of milk with avidity. At this time the treatment with calabar bean was begun. In the evening when the urine was drawn, he had a spasm, attended with slight opisthotonos. For the next three days he had frequent spasms, but no opisthotonos, but on the fourth and fifth the spasms were severe, and attended with trismus.

Aug. 31st.—The breathing was so hard that the calabar bean had to be stopped for an hour, and the whisky which had been given continuously, had to be increased. The symptoms continued in this way till Sept. 8th, the patient feeling sometimes stronger and at others weaker. During all this time, though the cramps came on frequently, they were not generally severe. The opisthotonos, when it did occur, was slight, and he was almost always able to swallow and take nourishment, until Sept. 8th, when a little after nine p. m., he was seized with a spasm, and as the physician had been called away, and hence was not able to treat the attack in time, he died.

I have given this full account: 1st, because it was such a remarkably marked case of tetanus, the symptoms being so full, and following each other with such regularity; 2d, because, as I had to record a death in this case, I hoped to show that the nerve stretching, instead of doing harm, did good, as I think no one will doubt, should the details be carefully studied. These, of course, in a paper of this kind, I have been unable to give. A statement of Dr. Callender, of St. Bartholomew, which I shall give later, will corroborate this opinion, namely, that the stretching, though it did not cure, was of service, and should have been tried. In conclusion of this case I give the opinion of Dr. J. C. Cameron, who attended the patient throughout. "Could I have been with him after 9 p. m., the fatal termination might have been warded off, for he had come safely through very much worse spasms than this, when he was far more prostrated. He was so much stronger and better than he had been for days, that we were all confident that he would recover. The inattention and culpable neglect of one of the attendants is no doubt, in a great measure, answerable for the poor fellow's death. It was ascertained, when it was too late, that his whisky, upon which we relied to maintain the heart's action and counteract the depressing effects of the bean, had not been given to him regularly nor in the quantity prescribed, as a portion of it had been taken by one of the attendants. This unfortunate affair is much to be regretted, as the case was progressing so favorably, and bid fair to be a triumph for nerve stretching and calabar bean."

In the *Medical Times and Gazette* for Sept. 15th, 1877, in an article on this subject, there is slight reference to an operation, by Dr. Patruban, which simply announces that in 1872 he laid bare and stretched the sciatic nerve for sciatica with great amelioration of symptoms. But as I cannot find the case in any of the journals in the Boston medical library, which, thanks to the courtesy of Dr. Chadwick, I have had the liberty of using, I am unable to give the details, and must content myself with a bare statement of the fact. There is another reference to a case of Von Nussbaum, in 1875, in which he laid bare and stretched the tibial and peroneal nerve for reflex epilepsy, complete cures resulting; also to one of Vogt's, in which he, in 1876, stretched the inferior dental for neuralgia, and the patient was cured. The same article reports that Kocher stretched the tibial nerve for rheumatic tetanus, but does not give any result. The same excuse that I have just made, must apply here. The article is a résumé of a work on nerve stretching by Dr. Paul Vogt, called Die Nervendehnung als Operation in der chirurgischen Praxis. To the cases which we have collated, it adds three more of tetanus, in which two were cured by him after operation. The first time that this operation was performed in America was, I think, on the 15th of May, 1876, by Dr. Edmund Andrews, surgeon to the Mercy Hospital and professor of surgery in the Chicago Medical College. For this operation, as for two years

I was his assistant in his private practice, I had the pleasure of giving the anæsthetic.

The patient, a sailor, about a year before the operation, fell from a yard to the deck of a ship, fracturing thereby two ribs and his right leg, causing total loss of motion and sensation in both the inferior extremities. The sensation afterwards returned, as did the motion, but this was perverted. When he was admitted to the Mercy Hospital, he complained of cramps and the most excruciating pains when his legs were straightened. His chief symptom was constant tonic spasm of the adductors of the thigh. This was so remarkably developed, and pressed the knees so painfully together, that he was obliged to wear a cushion between them to relieve the pressure; again, as these cramps came on when the legs were straightened, he was in the habit of using, to prevent this, a harness of his own construction and device, resembling that used in lithotomy, as the legs in this case were drawn out involuntarily. Another peculiar feature in this case was, that if the end of the penis was lightly touched with the finger, the thighs opened freely for a minute, though they closed again afterwards. On the fifteenth of May, the patient was anæsthetized. The patient was soon fully under the influence of the ether, that is, as far as one can judge by the ordinary signs, such as absence of reflex phenomena on pinching the eyelids, and making slight incisions with the knife. Of none of these irritants was he conscious, but the minute that his legs were straightened out, he became aroused, and another spasm occurred. This was tried twice in succession, and both times with the same result. So, that finally, to keep him quiet for the operation, I was obliged to keep him anæsthetized to such a degree, that he was in danger of being asphyxiated, in fact, one man had to drag out his tongue with a tenaculum during the entire operation. Dr. Andrews cut down, exposed, and stretched quite strongly the great sciatic and anterior crural nerves of the *left thigh*, then the wound was dressed with antiseptic dressings. The patient was relieved, strange to say, in the *right leg*. On the 24th, the operation was repeated, but on the *right leg*, and the patient was very much eased in his *left*. He was kept in the hospital until the 6th of September, when he was discharged, not cured, but very decidedly improved. And this improvement continued, as a few months afterwards Dr. Andrews received a letter from him in Liverpool, saying that though he had slight cramps occasionally, he still had been able to work his passage across. There are three noticeable points in this case that I cannot find in any other: 1st. Touching the penis caused the thighs to open. 2d. The phenomena under ether. 3d. Stretching the nerves of the left leg helped the right, and vice versa.

We have here sixteen cases with four deaths, and one case which I must put down as doubtful, thus giving us a mortality of 25 per cent. Although these cases are too few to justify any definite conclusions, and more must be collected, and careful statistics compiled before we can come to any exact determination, yet even from these, we are able to draw inferences, and these in favor of this operation, which has been tried in many affections of the nervous system such as neuralgia, paraplegia, trismus, etc. In all it has relieved, and in some cases, even in those cases in which death did occur, it made the death less painful and at the same time prolonged life. Again looking at the cases of mortality, we enquire if it was due so much to the operation as to the disease. In one of the cases the patient had hemiplegia for 37 years, then pain began suddenly to come on showing that some change was taking place in the system, and it is not unreasonable to suppose that this pain was the forerunner of death, and that the operation, while it was unable to prevent, at least did not produce the fatal result. The other cases were those of severe tetanus, a disease which when severe, generally causes death; we may therefore conclude that this operation of nerve stretching if not perfectly safe, is at least not dangerous.

Dr. Callender says there is no reason to fear trouble in nerve stretching, on the side of the nerve centre, as from the cases cited by himself, Nussbaum, and Billroth, it is proved that the nerves freed from their surrounding at the point from which traction is made, will tolerate stretching well. Nor on the other hand, is there any risk of disturbing the junction at the peripheral end, by this operation, nor is the nutrition interfered with, by isolation, as such nerves as the ulnar, musculo-spiral and ischiatic have been isolated and yet no harm has arisen, so that we may be sure that the exposure and stretching of nerves will not be followed by any bad result. In regard to the theory of this operation he says: the relief is not from any stimulating effect on the nerve fibres, because it is not to be supposed that the violent traction made in these cases can have a stimulating effect. He thinks that in some cases the laying bare of nerves and the freeing it from the abnormal adhesions with the adjacent structures, may be a source of relief as is shown in those cases in which great pain is felt in joints, from the straining of the adhesions which are often slight, but very sensitive to stretching. The relief is given by the complete tearing across of those adhesions, and here it is reasonable to suppose, that equal relief might follow the tearing off of the adhesions fixing the nerve, by the operation of nerve stretching. Though it is possible for this occasionally to happen, yet such an occurrence is probably exceptional, for the reason that adhesions, if they should exist, are most likely to be found around the distal end in relation to the scar, and would practically be destroyed as far as the conveying sensory impressions was concerned, by freeing the nerve from its surroundings, immediately above the cicatrix, either by dividing or cutting a portion of it. And yet this rarely succeeds, except in those cases, in which a single nerve is alone affected. Therefore he advances as the most plausible theory, that the stretching is of use by benumbing the nerve for a short time, not paralyzing it, because in the cases in which continuous nerves have been operated upon, motion and sensation have been retained in the parts to which the nerve was distributed, deadened indeed, for a short time, but not even temporarily lost. By this benumbing process the transit of abnormal impressions conveyed along the fibres of the nerves may be broken, and in the interval gained, the centres may reassume their natural state, just as in the operation proposed by Brown Séquard, in which the nerve, when exposed is washed repeatedly with ether, and thus rendered for many days quite incapable of transmitting any irritation. What is needed in these cases may be, first the freeing of the nerves from the conditions which caused the local irritation, and in the second place the temporary benumbing of the nerve trunk, in order to interrupt the chain of impressions which have habitually passed into and through the nerve centre.

Dr. Richard Wolseley mentions an interesting case of benumbing a nerve temporarily, by ether. The patient suffered from a fibroid tumor of the uterus, which was so large as to cause pressure on the sciatic, producing intense and almost continuous pain down the thigh and leg, for the relief of which sedative applications were ineffectual. At last he tried the application of the ether spray down the course of the nerve in the thigh which was done for about two minutes and gave immediate relief, and the pain did not return for ten days ; since that time he has tried the same in sciatica, and advised others to do so, and in every case relief was afforded.

In the *Boston Medical and Surgical Journal*, August 30th, 1877, Dr. James J. Putnam, mentions two cases, in which the patients had been suffering for months from neuralgia, and were cured by this operation ; and says there was no reason to believe that the nerves were bound down by any adhesions which were broken by the stretching, and the operation must be looked upon as a means of exerting a profound impression upon the affected part of the nerve centres.

Valentin, after a series of experiments, arrived at the following conclusions :

1st. Stretching lengthens the primitive fasciculi and decreases their calibre, and the nerve sheath presses upon the medullary substance. Electric excitability is not much interfered with, *provided* the stretching be not too considerable.

2d. If the stretching has not been carried too far, a nerve will quickly recover itself; the time required for recovery is in direct ratio to the weight applied and the length of time during which it was applied. If the stretching has been carried to such a point that no further excitability can be produced, it may nevertheless recover perfectly after a sufficient period of rest.

3d. The microscopic examination of nerves which have been stretched to their utmost, fail, as a rule, to discover anything abnormal, except that the medullary substance seems in places to be separated from the nerve sheath, just where the rupture of the sheath seemed to be commencing.

In closing this article, I cannot do better than to quote liberally from the before mentioned article in the *London Medical Times and Gazette*, as follows: "When this operation is compared with the only other operative procedure which is open to the surgeon, it stands out in prominent and favorable contrast; for neurotomy, hitherto employed, brings about its mechanical interruptions in nerve-conduction by means of solution of continuity, and on this account, therefore, the operation has, for the most part, only been applied to sensory nerves; for division of a motor nerve would be followed by immediate paralysis of muscles or even limbs supplied by this nerve. Further, the disease may be either central or peripheral, and division of the nerve, under any circumstances, could hardly affect a central cause, and hence in many cases would be contra-indicated. Stretching is indicated in neuralgia:

1st. In combination with neurotomy, when we have a case of peripheral origin affecting a purely sensory nerve, when all other therapeutical means have failed, and it is a case in which no special local means are indicated, such as removing a scar, foreign body, or morbid growth. By means of this combination we get rid of the peripheral irritation, or at least get an interruption of communication with the nervous center, and also a diminution of the irritability in the course of the entire nerve trunk since the action of nerve stretching goes much further in this direction than simple division of the nerves; also from the circulatory change which results in an alteration in the nutrition of the nerve. In this operation the blood vessels within the sheath become stretched and displaced, as is shown by the tortuosity and marked dilatation of the vessels supplying the nerves, while an altered condition of the vessels within the nerve-trunk does not seem to take place.

2d. It should be tried, in those cases of neuralgia, depending upon the mixed nerves, after all causes of local irritation have been removed, such as scars, etc., and all therapeutic measures tried.

In epilepsy, depending on some appreciable or fairly obvious injury of a peripheral nerve distribution.

In traumatic tetanus, but here should be no delay, until other means have failed, but the operation should be immediate.

To sum up, this operation may be needed when the symptoms are exalted sensibility, and disturbed function, due to disturbed blood circulation at the periphery.

